# Studies on Human Cultured Fibroblasts and Cutaneous Squamous Cell Carcinomas Suggest That Overexpression of Histone Variant H2A.J Promotes Radioresistance and Oncogenic Transformation

**DOI:** 10.3390/genes15070851

**Published:** 2024-06-27

**Authors:** Benjamin M. Freyter, Mutaz A. Abd Al-razaq, Markus Hecht, Christian Rübe, Claudia E. Rübe

**Affiliations:** Department of Radiation Oncology, Saarland University Medical Center, 66421 Homburg, Germanymarkus.hecht@uks.eu (M.H.);

**Keywords:** histone variant H2A.J, ionizing radiation, radiation-induced senescence, senescence-associated heterochromatin foci (SAHF), radioresistance, WNT signaling, oncogenic transformation

## Abstract

**Background:** Cellular senescence in response to ionizing radiation (IR) limits the replication of damaged cells by causing permanent cell cycle arrest. However, IR can induce pro-survival signaling pathways that reduce the extent of radiation-induced cytotoxicity and promote the development of radioresistance. The differential incorporation of histone variant H2A.J has profound effects on higher-order chromatin organization and on establishing the epigenetic state of radiation-induced senescence. However, the precise epigenetic mechanism and function of H2A.J overexpression in response to IR exposure still needs to be elucidated. **Methods:** Primary (no target, NT) and genetically modified fibroblasts overexpressing H2A.J (H2A.J-OE) were exposed to 20 Gy and analyzed 2 weeks post-IR for radiation-induced senescence by immunohistochemistry and immunofluorescence microscopy. Transcriptome signatures were analyzed in (non-)irradiated NT and H2A.J-OE fibroblasts by RNA sequencing. Since H2A.J plays an important role in the epidermal homeostasis of human skin, the oncogenic potential of H2A.J was investigated in cutaneous squamous cell carcinoma (cSCC). The tissue microarrays of cSCC were analyzed for H2A.J protein expression pattern by automated image analysis. **Results:** In response to radiation-induced DNA damage, the overexpression of H2A.J impairs the formation of senescence-associated heterochromatin foci (SAHF), thereby inhibiting the SAHF-mediated silencing of proliferation-promoting genes. The dysregulated activation of cyclins and cyclin-dependent kinases disturbs cell cycle arrest in irradiated H2A.J-OE fibroblasts, thereby overcoming radiation-induced senescence. Comparative transcriptome analysis revealed significantly increased WNT16 signaling in H2A.J OE fibroblasts after IR exposure, promoting the fundamental mechanisms of tumor development and progression, including the activation of the epithelial–mesenchymal transition. The quantitative analysis of cSCCs revealed that undifferentiated tumors are associated with high nuclear H2A.J expression, related with greater oncogenic potential. **Conclusion:** H2A.J overexpression induces radioresistance and promotes oncogenic transformation through the activation of WNT16 signaling pathway functions. H2A.J-associated signatures may improve risk stratification by identifying patients with more aggressive cSCC who may require radiotherapy with increased doses.

## 1. Introduction

Cutaneous squamous cell carcinoma (cSCC) is the most common form of skin cancer and arises from epidermal keratinocytes, usually basement membrane-bound epithelial cells [1]. The gradual transition from healthy epidermal keratinocytes to precancerous lesions and subsequent cSCC involves cumulative genetic and epigenetic changes in the signaling pathways that control proliferation, differentiation, and aberrant interactions with the microenvironment and immune surveillance [2,3]. Malignant transformation commonly leads to the disruption of cell cycle control mechanisms and terminal differentiation programs of keratinocytes, and thus disturbs the formation of functional skin permeability barrier [4]. In healthy skin, normal keratinocytes are characterized by stable cell–cell junctions, apical–basal polarity, and close interactions with the basement membrane. In response to intrinsic or extrinsic signals, epithelial–mesenchymal transition (EMT) is triggered leading to the suppression of the epithelial and acquisition of the mesenchymal properties, an important characteristic of tumor progression in cSCC [5]. The overall frequency of metastasis in cSSC is relatively low. However, the recurrence of cSCC indicates an aggressive tumor with higher metastasis rates leading to significant morbidity and mortality [1]. Current management should be improved to more accurately identify the tumors most likely to relapse and metastasize following surgical removal and/or radiotherapy. Histopathological staging for cSCC is not optimal for predicting accurate outcomes, but recent studies suggest that genetic and epigenetic signatures may improve risk prediction [4]. Moreover, tumor stroma containing cancer-associated fibroblasts also exert important pro-tumorigenic functions by producing pro-inflammatory mediators [6,7]. Tumor-associated fibroblasts can support tumor progression and metastasis by fostering tumor cell growth, influencing immune responses, remodeling ECM, and promoting the development of therapeutic resistance [6,7].

Chromatin organization is the template for transcriptional regulation and chromatin-based modifications form the molecular basis for the epigenetic regulation of fundamental cellular functions [8]. The precise configuration of chromatin, including the structure, stability, and positioning of nucleosomes, as well as their three-dimensional folding, has a decisive impact on genome accessibility for the binding of transcription factors and thus for epigenetic regulation. Chromatin organization occurs at multiple levels and contributes to the correct temporal and spatial progression of transcription programs that are essential for fundamental cellular functions, such as proliferation and differentiation, and therefore has particular importance for oncogenesis [9]. Among epigenetic mechanisms, the replacement of replication-coupled canonical histones with histone variants can be associated with dramatic chromatin remodeling [10,11], contributing particularly to the plasticity of the epigenome [10].

The cell division cycle is regulated by complex control mechanisms to ensure a unidirectional transition through the cell cycle, once prompted by external stimuli and permitted by internal checkpoints [12]. The tasks of replicating the whole genome during S-phase and accurately separating chromosomes and cell organelles into daughter cells during mitosis require multiple specialized proteins encoded by cell cycle-dependent genes. Growth stimuli elicit cellular signaling cues that lead to the activation of cyclins and cyclin-dependent kinases (CDKs) that enable cell cycle entry and coordinate precisely timed cell cycle progression [12]. The degradation of specific cell cycle proteins through the ubiquitin system provides an additional layer of control [13]. Importantly, these control mechanisms regulate one another and generate feedback loops and redundancies to provide an almost fail-safe system of progression from G1 through S-/G2-phases and into mitosis followed by cell division. Disturbances in this complex system, including epigenetic dysregulation, can lead to uncontrolled cell proliferation and thus may contribute to tumor progression.

Cellular senescence is a stable proliferation arrest that can be triggered by telomere shortening (replicative) or by oxidative, genotoxic, and other cellular stresses (premature senescence). Senescence has emerged as an important tumor-suppressing mechanism to prevent the further proliferation of pre-malignant cells [14]. Senescent cells are characterized by profound changes in morphology and metabolism as well as by extensive chromatin reorganization in the nucleus [15]. Previous studies have shown that histone 2A subfamily member H2A.J (gene encoding H2A.J: H2AFJ) is progressively incorporated into chromatin during replicative and premature senescence and regulates the gene expression of pro-inflammatory mediators as part of the senescence-associated secretory phenotype (SASP) [16,17]. During senescence development, the most striking nuclear phenotype is the formation of heterochromatin domains, called senescence-associated heterochromatic foci (SAHF) [15]. SAHF are visible as clear DNA foci after diamidino-2-phenylindole (DAPI) staining and reflect condensed chromatin, enriched with heterochromatic histone modifications [18]. These epigenetic marks repress the transcription of key proliferation-related genes and link SAHF to the tumor-suppressive cell cycle arrest of senescent cells [15]. Previous work has highlighted the importance of histone variant H2A.J in the context of the epidermal homeostasis of the skin, both during the physiological aging process and acute stress response following exposure to ionizing radiation (IR) [19,20]. Following IR exposure, the incorporation of H2A.J crucially influences the chromatin structure locally and globally, and thus the binding of transcription factors, resulting in the modulation of gene expression [21]. Previous work on skin irradiation has shown that the increasing incorporation of H2A.J after IR exposure results in suppressed proliferation and that keratinocytes expressing H2A.J have lost their ability to proliferate [19]. However, recent studies have shown that this radiation-induced cell cycle arrest may be overcome by strong H2A.J overexpression [21].

The disruption of chromatin homeostasis by core and linker histone alterations is a hallmark of cancer and often promotes oncogenic transformation [22]. Targeting cancer-associated epigenetic changes in both tumor cells themselves and stromal cells surrounding the tumor can potentially be used for therapeutic interventions. In addition, epigenetic marks can serve as biomarkers for the diagnosis and prognosis of tumors, but also for predicting their treatment response. Due to the particular importance of H2A.J in radiation-induced senescence, the potential oncogenic effects of H2A.J overexpression in human fibroblasts were investigated before and after IR exposure at the transcriptome level. Since H2A.J has an important impact on skin physiology, H2A.J protein expression in the tumor samples from patients with cSCCs was precisely quantified and correlated with tumor stage and grading. The aim of this work was to investigate the functional significance of H2A.J overexpression for the development of radioresistance and tumor progression.

## 2. Materials and Methods

Cultured fibroblasts: Primary WI-38 human fibroblasts were obtained from ATCC. Immortalized WI-38 hTERT-fibroblasts were genetically modified to prepare H2A.J-overexpressing (H2A.J-OE) fibroblasts, and compared to non-targeted controls (NT), as described previously [16]. Stable fibroblast populations were cultured at 5% O_2_ and 5% CO_2_ in MEM (Invitrogen, Karlsruhe, Germany) with 10% fetal bovine serum, 1 mM sodium pyruvate, 2 mM L-glutamine, 0.1 mM non-essential amino acids, and 1% penicillin/ streptomycin. A total of 1 µg/mL doxycycline was added to the medium for 1 week prior to IR to express shRNA sequences to overexpress H2A.J. The fibroblasts were homogenously grown and used for experiments once 90% confluency was achieved.

Radiation exposure: The fibroblasts were exposed to IR using the linear accelerator Artiste™ (Siemens, Munich, Germany) (6-MV photons; dose-rate 2 Gy/min). The cells were analyzed 2 weeks following IR with 20 Gy (2w post-IR) and compared to non-irradiated controls (non-IR).

Immunofluorescence microscopy: The fibroblasts were fixed with 4% paraformaldehyde and permeabilized with 0.5% Triton X-100, washed with 0.1% Tween^®^-20, and incubated overnight with a primary antibody (anti-H2A.J, ActiveMotif, Waterloo, Belgium; anti-p21, Abcam, Berlin, Germany) followed by Alexa-Fluor^®^488 or Alexa-Fluor^®^568 secondary antibody (Invitrogen, Karlsruhe, Germany). Subsequently, the cells were mounted in a VECTAshield™ mounting medium with 4′, 6-diamidino-2-phenylindole (DAPI, Vector Laboratories, Burlingame, CA, USA). Fluorescence images were captured with a Nikon-Eclipse Ni fluorescence microscope equipped with a Nikon DS-Qi2 camera (Nikon, Düsseldorf, Germany). For evaluating H2A.J- and p21-positivity, at least 200 cells were captured for each sample (positive cells in %).

Immunohistochemical analysis: Following 5 min fixation with 2% paraformaldehyde and 0.2% glutaraldehyde, the cells were incubated with X-Gal staining solution (AppliChem GmbH, Darmstadt, Germany) at 37 °C overnight. After 30 s of methanol incubation, the dried samples were permeabilized with 0.2% Triton X-100 and washed with 1% BSA. The samples were blocked with 4% BSA for 1 h, followed by overnight incubation with H2A.J primary antibody. Incubation with Dako immunoglobulin/bioatinylated secondary antibody (Agilent, Waldbronn, Germany) was followed by Vectastain ABC Peroxidase standard (Vector, Burlingame, CA, USA) and SIGMAFAST™ 3.3’Diaminobenzidine (Merck, Darmstadt, Germany) incubations, respectively. The samples were finally mounted in Dako Faramount Mounting Medium (Agilent, Waldbronn, Germany).

Transcriptome analysis by RNA-sequencing: RNA extraction, library preparation, and paired-end (2 × 150bp, 30M reads per sample) sequencing using NovaSeq (Illumina, San Diego, CA, USA) were performed by GENEWIZ Germany GmbH (Leipzig, Germany). Subsequently, raw reads were trimmed using Cutadapt (v3.5) to cut off the low-quality ends of the reads (below Phred quality score of 20) and a minimum read length of 50 was set. The data quality was inspected before and after trimming using FastQC (v0.11.3) [23]. The sequences were aligned to the human genome sequence GRC38.p13 [24] using the Burrows–Wheeler Alignment Tool (v0.7.17) [25] and counted with HTSeq (v0.13.5) [26]. Subsequently, a differential expression analysis was performed using DESeq2 (v1.34.0) [27].

Immunohistochemical staining of H2A.J in cSCCs: Tissue microarray (TMA) slides with 76 cores (1.5 mm diameter) of formalin-fixed primary SSCs embedded and arrayed in paraffin for high-throughput immunohistochemistry staining were purchased from TissueArray.Com LLC (#SK802c; Derwood, MD 20855, USA). The patients’ age and gender, as well as anatomical tumor location are described in Table 1. For each tumor sample, the TNM stage, differentiation status, and tumor stage were indicated by experienced pathologists. For IHC studies, two TMA slides were stained to ensure consistent antigenicity and combined with H&E stains. After dewaxing in xylene and rehydration in decreasing ethanol concentrations, antigen retrieval was performed in citrate buffer, and sections were incubated with anti-H2A.J antibody (1:500, Abcam, Cambridge, UK, Cat. no. ab54210) followed by biotin-labeled antibodies (Dako, Glostrup, Denmark). Staining was completed by incubation with 3,3′-diaminobenzidine and substrate chromogen. Finally, the sections were counterstained with hematoxylin and mounted with Aqueous Mounting Medium (Dako, Glostrup, Denmark).

Digital image analysis of H2A.J expression in cSCCs: Whole digital images were obtained from H2A.J-stained TMA using the slide scanner microscope Axioscan 7 (ZEISS, Oberkochen, Germany). The HALO^®^ module TMA add-on (version 3.5.3577; IndicaLabs, Albuquerque, NM, USA) with the automated segmentation of each tumor core was used to enable an efficient workflow of the TMA analysis. According to nuclear staining intensities, a threshold value was defined for chromogenic H2A.J-signal to detect H2A.J+ cell nuclei. The H2A.J+ cells were automatically identified and segmented, and the percentages of H2A.J+ cells relative to total cell numbers were calculated for each tumor lesion.

Statistical analysis: GraphPad Prism (version 9.4.1, GraphPad Software, San Diego, CA, USA) was used to collect and analyze data. The data were presented as the mean of three experiments ± SEM. One-way analysis of variance (ANOVA) with Dunnett’s multiple comparisons test was used for comparison among the different groups. A *p*-value of <0.05 was considered statistically significant, <0.01 as statistically highly significant, and <0.001 as extremely statistically significant. Significant statistical differences compared to non-irradiated controls or between cell lines (asterisks with square brackets) are presented in the figures as * (*p* < 0.05), ** (*p* < 0.01), and *** (*p* < 0.001).

## 3. Results

### 3.1. H2A.J Overexpression Leads to Overcoming Radiation-Induced Senescence

**Figure 1:**
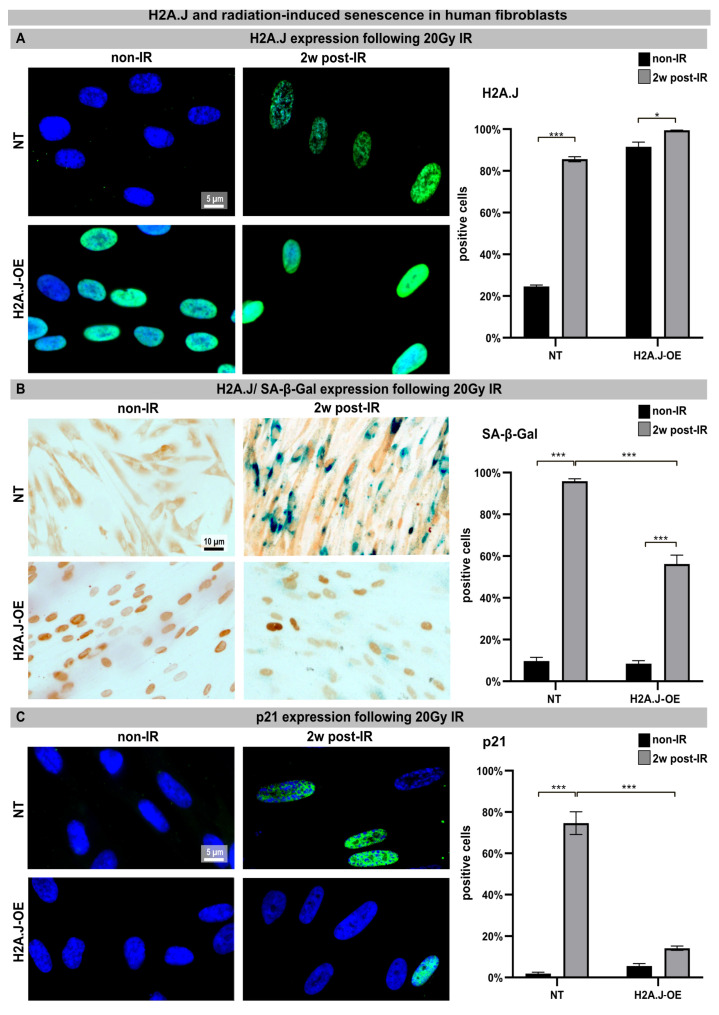
(**A**) H2A.J expression: The IFM micrographs of the NT and H2A.J-OE fibroblasts show the H2A.J+ cells at 2 weeks post-IR (2w post-IR) compared to the non-irradiated fibroblasts (non-IR). The adjacent graph shows the quantification of the H2A.J+ cells. (**B**) H2A.J expression and SA-β-Gal staining: IHC micrographs show the H2A.J and SA-β-Gal staining of the (non-) irradiated NT and H2A.J-OE fibroblasts. The adjacent graph shows the quantification of the H2A.J+ and SA-β-Gal+ cells. (**C**) p21 expression: IFM micrographs show the p21 staining of the (non-) irradiated NT and H2A.J-OE fibroblasts. The adjacent graph shows the quantification of the p21+ cells. The data are presented as the mean of three experiments ± SE. Significant statistical differences were marked by asterisks with square brackets: * *p* < 0.05; *** *p* < 0.001.

Confluent NT and H2A.J-OE fibroblasts were exposed to an IR dose of 20 Gy. The expression of H2A.J was examined by immunofluorescence microscopy before (non-IR) and two weeks after IR exposure (2w post-IR). In non-irradiated NT fibroblasts, there was either no or only minimal staining for H2A.J. However, H2A.J-OE fibroblasts showed strong H2A.J staining, even without IR. Following IR exposure, both types of fibroblasts exhibited intense pan-nuclear staining for H2A.J (Figure 1A, left panel). The quantitative analysis revealed that the percentage of the H2A.J+ cells increased from ≈20% in the non-irradiated NT fibroblasts to ≈80% in the irradiated NT fibroblasts (Figure 1A, right panel). Moreover, while ≈80% of the H2A.J-OE fibroblasts displayed intense H2A.J staining without IR exposure, this proportion of the H2A.J+ cells increased to nearly 100% following the IR exposure (Figure 1A, right panel). These results demonstrate the successful overexpression of H2A.J so that its functional significance in this cell system can be investigated. Senescent cells are commonly identified using senescence-associated β-galactosidase (SA-β-Gal) as a biomarker. Double-staining techniques were established to detect H2A.J and SA-β-Gal (nuclear brown versus cytoplasmic blue signal) simultaneously (Figure 1B, left panel). The activity of SA-β-Gal in the non-irradiated NT and H2A.J-OE fibroblasts was generally low, with only ≈10% of the cells showing SA-β-Gal positivity. However, after the IR exposure, the NT fibroblasts exhibited increased expression of lysosomal β-galactosidase protein, resulting in a significant increase in the proportion of SA-β-Gal+ cells to ≈95% at 2w post-IR. This suggests that these fibroblasts properly enter the senescent state (Figure 1B, right panel). On the other hand, the H2A.J-OE fibroblasts showed only a slight induction of SA-β-Gal in ≈50% of the cells at 2w post-IR, indicating that large proportions of these cells failed to enter senescence (Figure 1B, right panel). To determine cell growth arrest associated with radiation-induced senescence, we examined the levels of cyclin-dependent kinase inhibitor proteins, specifically p21^Cip1/Waf1^. To validate our observation of reduced senescence induction in the H2A.J-OE fibroblasts following the IR exposure, we performed p21 staining (Figure 1C, left panel). The non-irradiated NT fibroblasts exhibited minimal p21 levels (≤3%), whereas the proportion of p21+ cells in the NT fibroblasts increased to ≈80% following the IR exposure, indicating strong senescence responses (Figure 1C, right panel). Interestingly, the H2A.J-OE fibroblasts maintained low levels of p21 expression (14%) even after the IR exposure, suggesting that these cells are largely resistant to radiation-induced senescence (Figure 1C, right panel).

### 3.2. H2A.J Overexpression Leads to Altered Transcription Programs after IR Exposure

**Figure 2:**
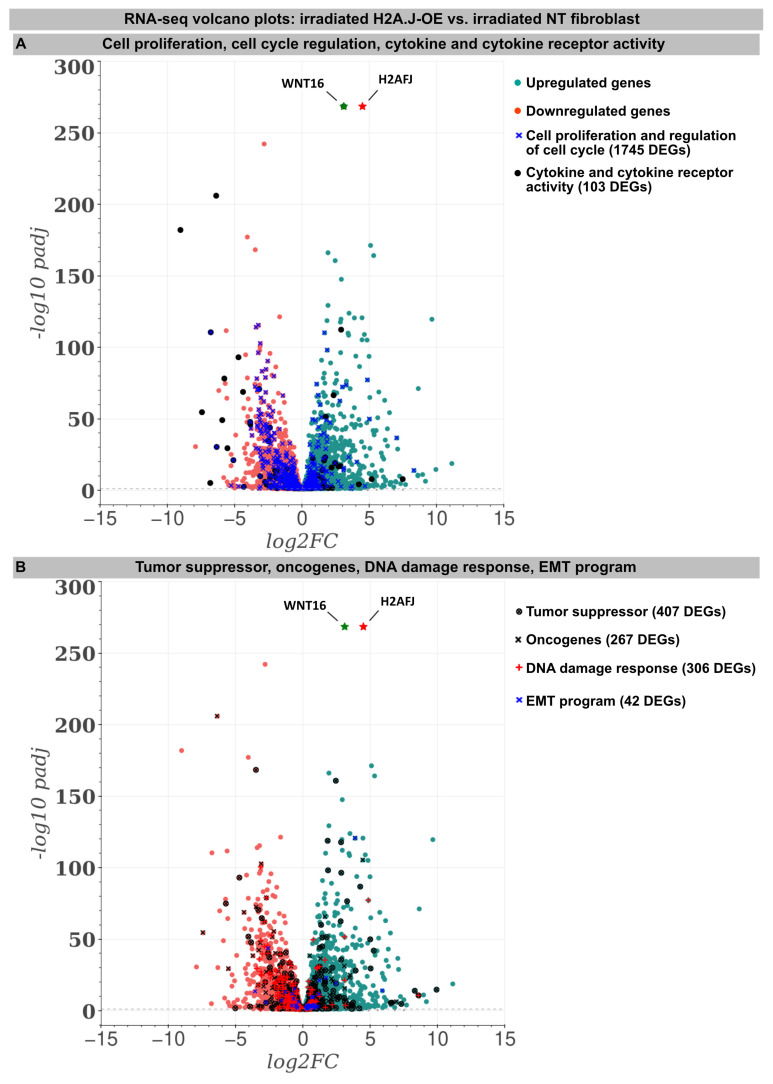
RNA-seq transcriptome analysis: The volcano plots of DEGs between the irradiated H2A.J-OE versus the irradiated NT fibroblasts. The scatterplots show the statistical significance (-*log10 padj*) versus the magnitude of change (*log2FC*). The genes are colored if they pass the thresholds for -*log10padj* and *log2FC*: Red/green dots denote down-/upregulated gene expression. DEGs classified according to different KEGG signaling pathways (**A**): cytokine and cytokine receptor activity, cell proliferation, and the regulation of cell cycle and (**B**): tumor suppressors and oncogenes, DNA damage response, and EMT program were visualized in comparison to other genes. Note that besides the H2AHJ gene, WNT16 was the most significantly upregulated gene.

For deciphering the stress response networks underlying radiation-induced senescence and accurately assessing the relative contribution of the H2A.J overexpression, protein-coding transcriptomes were determined by RNA-sequencing in the (non-)irradiated NT versus H2A.J-OE fibroblasts (Figure 2). Basically, all the (non-)irradiated fibroblast lines were compared with each other (NT IR vs. NT non-IR, H2A.J-OE IR vs. H2A.J-OE non-IR, and NT non-IR vs. H2A.J-OE non-IR), but in this work the focus was on the different radiation reactions of the H2A.J-OE (H2A.J-OE IR) versus NT fibroblasts (NT IR). Accordingly, the transcriptome analysis revealed 5681 differentially expressed genes (DEGs) between H2A.J-OE IR versus NT IR (of which 2775 were upregulated and 2906 downregulated). Gene expression profiles for many biological functions, classified according to the Gene Ontology, Tumor Suppressor Gene, and Oncogene databases, were fundamentally changed in the H2A.J-OE fibroblasts compared to the NT fibroblasts following the IR exposure (Figure 2). Many of these DEGs affected cell proliferation and cell cycle regulation (1745 DEGs), cytokine and cytokine receptor activity (103 DEGs), tumor suppressors and oncogenes (674 DEGs), DNA damage response (306 DEGs), as well as EMT program (42 DEGs). The H2A.J gene is expressed in non-irradiated, but even more pronounced in the irradiated H2A.J-OE fibroblasts (Figure 2). Of all the DEGs, WNT16 was the most significantly increased gene in senescent H2A.J-OE fibroblasts (Figure 2). WNT16 belongs to the WNT family, initially identified for its role in developmental processes and carcinogenesis. WNT signaling controls cell fate determination, cell proliferation, and migration processes necessary for proper tissue formation.

**Figure 3:**
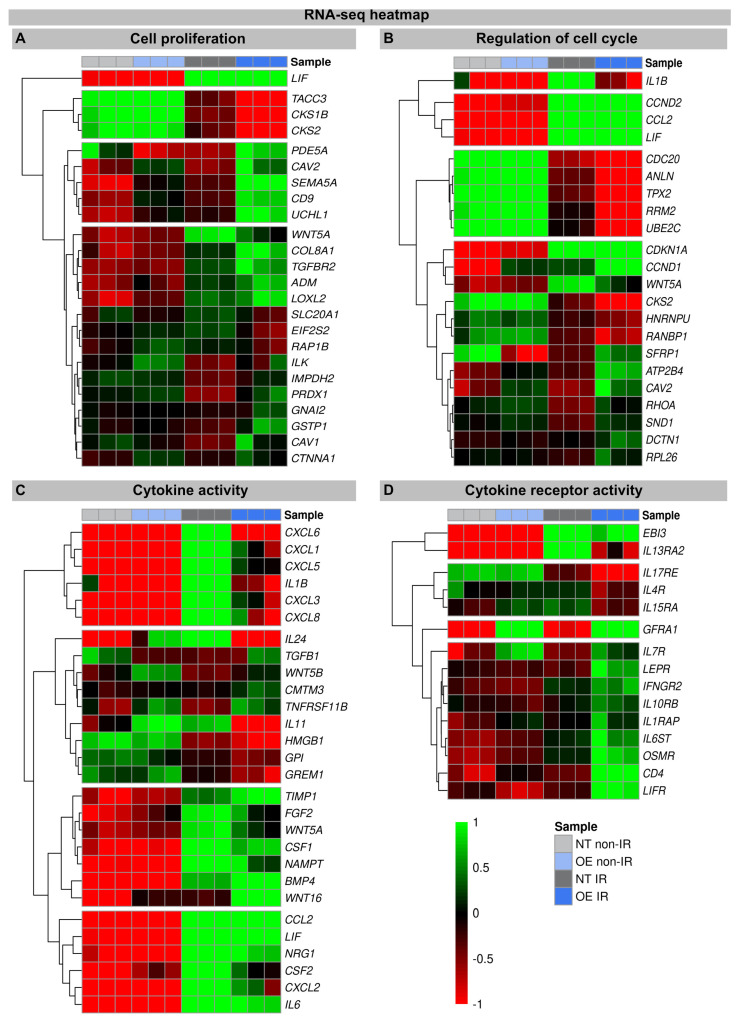
Heat map of RNA-seq expression data showing DEGs between (non-) irradiated H2A.J-OE versus NT fibroblasts. DEGs were classified according to different KEGG signaling pathways: (**A**): cell proliferation; (**B**): regulation of cell cycle; (**C**): cytokine activity; (**D**): cytokine receptor activity. DEGs were selected based on ±1-fold change and FDR < 0.05.

**Figure 4:**
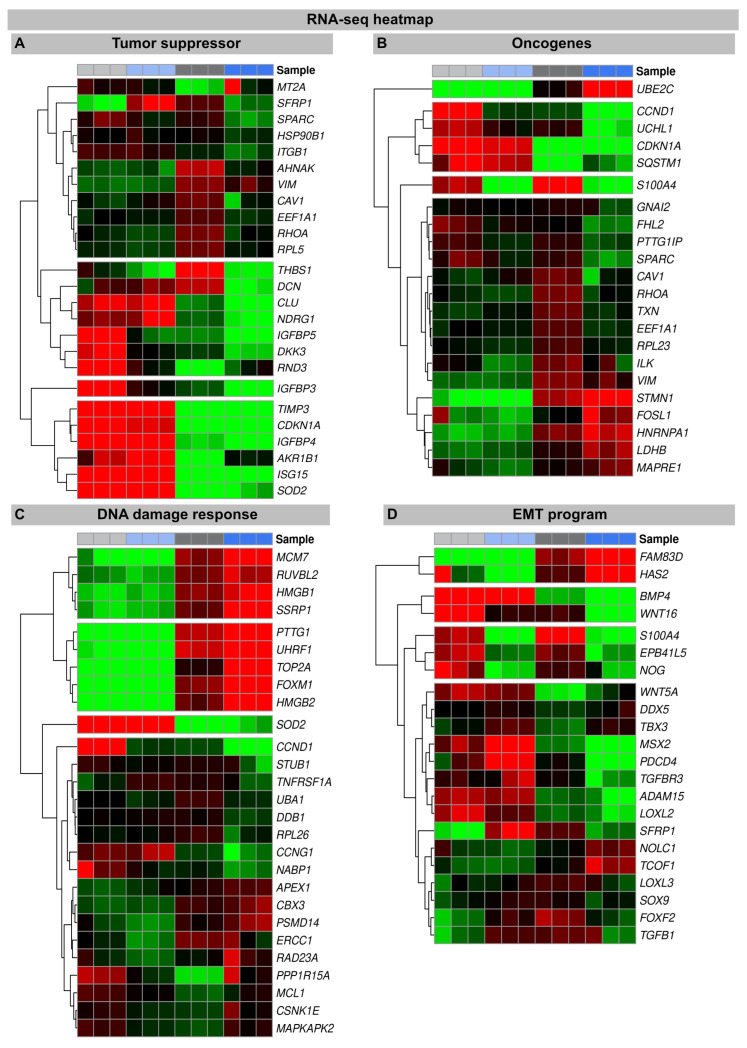
Heat map of RNA-seq expression data showing DEGs between irradiated H2A.J-OE versus irradiated NT fibroblasts. DEGs were classified according to following KEGG signaling pathways: (**A**): tumor suppressor; (**B**): oncogenes; (**C**): DNA damage response, (**D**): EMT program. DEGs were selected based on ±1-fold change and FDR < 0.05.

An increase in WNT16 is also observed in the NT fibroblasts following the IR exposure, but in the irradiated H2A.J-OE fibroblasts the increase in WNT16 was again 8.7-fold higher (Figure 2). Previous work has shown that WNT16 plays a crucial role downstream of p53 to modulate the expression of p21^WAF1^ in cells undergoing senescence [28]. Moreover, the WNT16 pathway has been associated with treatment-induced damage to the tumor microenvironment, promoting cancer therapy resistance, senescence reversal, and cellular transformation [29]. Overall, many components with oncogenic potential are expressed differently in H2A.J-OE IR versus NT IR, suggesting that H2A.J-OE versus NT fibroblasts respond differently to IR exposure (Figure 2). In our RNA-seq datasets, smaller differences in gene expression profiles were observed between the non-irradiated H2A.J-OE versus the NT fibroblasts regarding cell proliferation, cell cycle regulation, and cytokine and cytokine receptor activity (Figure 3), as well as DNA damage response and tumor suppressors and oncogenes (Figure 4). These results suggest that these cellular functions are largely independent of H2A.J expression under normal, unstressed conditions. Only for the EMT program, we observed significant differences between the non-irradiated H2A.J-OE versus the non-irradiated NT fibroblasts, some of which increased following the IR exposure (Figure 4B). Largest differences in gene expression profiles were observed between the non-irradiated and irradiated cell populations for both the NT and H2A.J-OE fibroblasts, reflecting the strong stress response caused by the IR exposure (Figure 3 and Figure 4).

#### 3.2.1. Proliferation and Cell Cycle Regulation

The heatmaps show multiple DEGs in the irradiated H2A.J-OE versus the irradiated NT, particularly with regard to cell proliferation and cell cycle regulation (Figure 3A,B). In-depth inspection confirms that most DEGs indeed encode for proteins with well-established roles in cell cycle regulation, including cyclins and cyclin-dependent kinases (e.g., CKS1B, CKS2, CCND2, CDKN1A, CCND1, and CKS2), mitotic spindle components (e.g., TACC3, CDC20, and HNRNPU), pre-replication complex assembly proteins (e.g., RRM2 and PDE5A), and kinesin and kinesin-related proteins (e.g., CDC20, ANLN, and TPX2). Corrupted cell cycle control systems can give rise to uncontrolled cell growth and thus may contribute to tumor development. Decisive differences between the H2A.J-OE versus the NT fibroblasts were observed for the cell cycle genes *Cyclin D1* (CCND1) and *CDC28 Protein Kinase Regulatory Subunit 2* (CKS2). These genes encode the binding partners of the catalytic subunits of cyclin-dependent kinases and exert regulatory effects on cell-cycle progression. Previous studies have shown that the aberrant expression of these cell cycle genes is closely associated with the development of radioresistance [30,31].

#### 3.2.2. Cytokine and Cytokine Receptor Activity

In these transcriptome signatures, key differences between the irradiated H2A.J-OE and NT fibroblasts were observed with respect to cytokines and cytokine receptors. As previously described, we observed clear differences in the expression of cytokines, chemokines, and growth factors between the irradiated NT and H2A.J-OE fibroblasts, which may modulate differently their inflammatory and proliferative behavior [16,21]. Our RNA-seq data sets showed a significant upregulation of cytokines (IL1B, IL6, IL11, and IL24), chemokines (CXCL1, CXCL3, CXCL5, CXCL6, and CXCL8), and growth factors (CSF1 and CSF2) in the irradiated versus non-irradiated NT fibroblasts. However, the cytokine expression of IL1B, IL-24, IL11, and CSF2 was clearly reduced in the irradiated H2A.J-OE fibroblasts. As previously described, WNT16 was increasingly expressed in the normal NT fibroblasts after the IR exposure as part of the senescence messaging secretome [28]. In replicative senescence, WNT16 activation is dependent on telomere shortening and the subsequent p53 activation [28]. Strikingly, this WNT16 expression was significantly increased in the H2A.J-OE versus NT fibroblasts during their stress-induced premature senescence. Indeed, our results showed that despite the upregulation of WNT16 in the H2A.J-OE fibroblasts, the p53/p21^WAF1^ signaling pathway was not fully activated (Figure 1A).

WNT16 signaling promotes the acquisition of mesenchymal cell properties, which can influence the migration and invasion behavior of epithelial cells associated with EMT [29]. Accordingly, significant changes in EMT networks were observed in H2A.J-OE versus NT fibroblasts (see below). Moreover, numerous cytokine receptors are increasingly expressed in the H2A.J-OE fibroblasts (but not in NT fibroblasts) following the IR exposure. Most of these cytokine receptors mediate their effects via Janus kinase (JAK) signal transducers and the activators of the transcription (STAT) signaling pathway to influence cell programs [32]. Basically, the control of cell proliferation integrates cytokine signals from various extracellular sources transmitted through cytokine receptors, indicating the close connections between the cytokine-activated signaling pathways and cell cycle regulatory mechanisms [33].

#### 3.2.3. DNA Damage Response

In senescent cells, the reorganization of chromatin with the formation of SAHFs contributes significantly to the stability of cell cycle arrest by silencing proliferation-promoting genes through heterochromatinization [34,35]. Our transcriptional analysis shows that, compared to non-irradiated fibroblasts, many components of the chromatin remodeling machinery (MCM7, RUVBL2, HMGB1, SSRP1, PTTG1, UHRF1, TOP2A, FOXM1, and HMGB2) were downregulated following the IR exposure. In previous work, we have already shown that the ectopic overexpression of H2A.J prevents the formation of SAHF so that proliferation genes cannot be silenced, and permanent cell cycle arrest cannot be achieved [21] (Figure S1). This mechanism potentially undermines the irreversibility of the senescent state and allows re-entry into the cell cycle. Accordingly, many chromatin-remodeling genes are more strongly downregulated in the irradiated H2A.J-OE fibroblasts versus the irradiated NT fibroblasts, potentially due to suppressed SAHF formation. In this context, HMGB proteins appear to be particularly important as the global sensors of cellular stress, because they determine the balance between cell death and survival responses, essential for cellular homeostasis and tissue maintenance. In the nucleus, HMGB proteins are, along with histones, the most important chromatin proteins, as they interact with nucleosomes to remodel compact chromatin, thereby organizing the binding of transcription factors and regulating gene expression [36].

#### 3.2.4. Tumor Suppressors and Oncogenes

To explore the oncogenic potential of H2A.J, we examined the RNA-seq datasets for the expression of tumor suppressors and oncogenes (Figure 4). Many tumor suppressors and oncogenes modulated by H2A.J play important roles in the regulation of energy metabolism (IGFBP3; IGFBP4; IGFBP5; AKR1B1; SOD2). During the tumorigenesis process, metabolic circuits are dramatically altered, which may reflect the reprogramming of energy metabolism. Growing tumors must meet the bioenergetic and biosynthetic demands of increased cell proliferation and survive environmental fluctuations in external nutrient and oxygen availability when tumor growth exceeds the delivery capacities of the existing vasculature [37]. Many of these metabolism-associated factors are upregulated following the IR exposure, e.g., IGFBP5 is more highly expressed in the irradiated H2A.J-OE versus the NT fibroblasts. The development and progression of cancer are multifaceted processes that involve the complex interplay of transformed cells with their microenvironment, which largely consists of extracellular matrix components. Accordingly, proteoglycans and glycoproteins are increasingly recognized as critical contributors in shaping tumor microenvironment by regulating the inflammatory and immune milieu [38]. Our transcriptome analysis showed different expression patterns of proteoglycans and glycoproteins (THBS1; DCN) in the non-irradiated versus the irradiated fibroblasts, but especially between the irradiated H2A.J-OE versus the NT fibroblasts. The cell cycle is tightly controlled by the activity of cyclin-dependent kinases (CDKs), their cyclin partners, and CDK inhibitors, as well as by the ubiquitin-proteasome system [13].

Our transcriptional analysis showed that many factors involved in this framework of cell cycle progression are dysregulated in the irradiated H2A.J-OE versus the NT fibroblasts (UBE2C; CCND1; UCHL1; CDKN1A). This correlates with our observation that H2A.J-OE fibroblasts do not enter radiation-induced senescence even following high-dose IR, in contrast to their normal counterparts.

#### 3.2.5. Epithelial-to-Mesenchymal Transition

During the EMT process, cell–cell junctions and cell–matrix attachments are disrupted, and the cytoskeleton is remodeled to enhance the mobility of cells through extracellular matrices. The execution of EMT with the reorganization of cell morphology and cytoarchitecture has been recognized as a crucial step in tumor progression [39]. Our RNA seq data showed that in response to high-dose IR, many genes of the EMT program were differently expressed in the H2A.J-OE versus the NT fibroblasts. Accordingly, differently expressed genes involved cell–cell junctions (PB41L5; ADAM15), cell–matrix attachments (HAS2; EPB41L5), cytoskeleton remodeling (FAM83D; S100A4), and cell movement through extracellular matrix (NOG; LOXL2) and developmental processes (WNT16; MSX2; BMP4). Collectively, our data suggest that upon H2A.J overexpression, dynamic changes in cellular and extracellular organization may lead to functional consequences in cell migration and invasion capacities following IR exposure.

### 3.3. H2A.J Protein Is Highly Expressed in Undifferentiated cSSC

**Figure 5:**
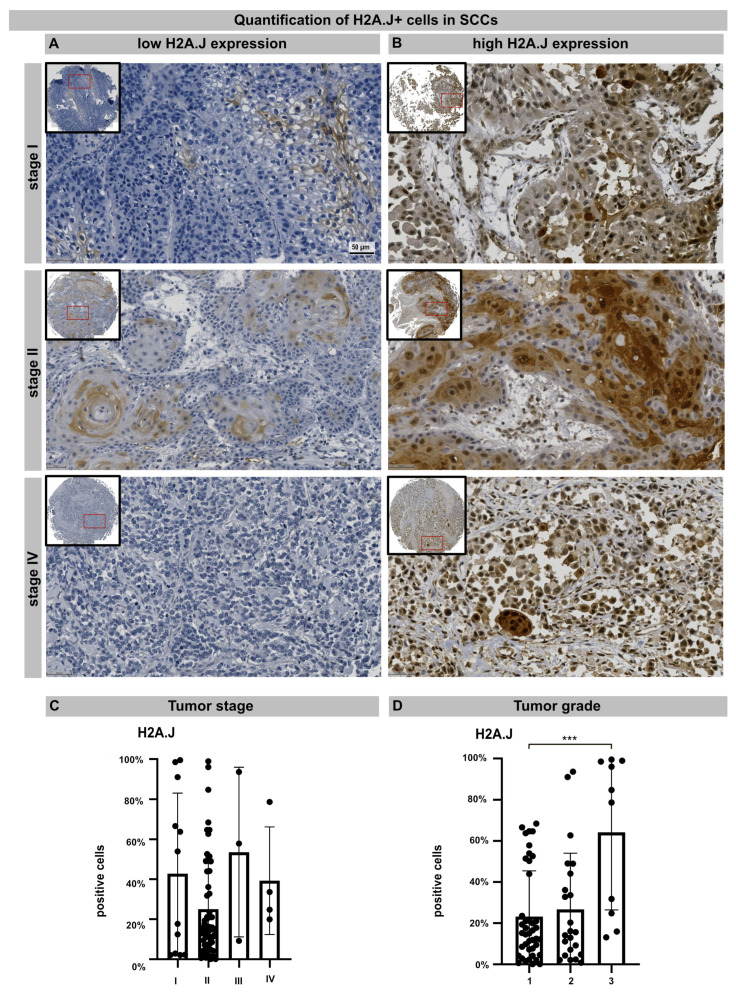
IHC micrographs showing low (**A**) or high (**B**) H2A.J expression in cSCC regardless of tumor stage. Quantification of H2A.J+ cells in relation to tumor stage (**C**) and tumor grade (**D**). Significant statistical difference is marked by asterisks with square brackets: *** *p* < 0.001.

TMA technology makes it possible to examine the pattern of protein expression in large numbers of samples using identical staining conditions. TMA containing 76 samples from primary cSSC derived from different anatomical sites were subjected to H2A.J immunohistochemistry. The age- and gender-associated distribution of patients, anatomical localization, and histologic features of primary cSCCs are presented in Table 1. After adapting the TMA map, all the samples were scanned and aligned automatically and the TMA cores containing insufficient tissue or artifacts were removed (Figure S2). For nuclear H2A.J positivity, a threshold was defined based on staining intensity, and the H2A.J+ nuclei were automatically segmented and quantified in annotated tumor areas. The various cSSC lesions exhibit remarkably wide histopathological diversity. Well-differentiated lesions revealed prominent keratinization and formed “pearl-like” structures, where the dermal nests of keratinocytes attempted to mature in a layered fashion. Moderately differentiated lesions of cSCC show much less organization and maturation with significantly less keratin formation. In different tumor samples, there is great variability in H2A.J expression with nuclear and cytoplasmic staining. According to our analyses, H2A.J expression in tumors showed no correlation with the patient’s age or gender, nor with skin location in relation to UV-light exposure (Supplementary Figure S1B). In primary cSCC, the size and depth of invasion are important determinants of the likelihood of tumor recurrence and metastasis. Typically, tumors that are either larger than 2 cm or have an invasion depth of more than 6 mm (affecting deeper cutis or even subcutis levels) have much more aggressive courses and significant risks of metastasis and recurrence. According to the TNM classification, most cSCCs examined have T1 (≤20 mm in maximum dimension) or T2 (>20 mm to ≤40 mm) tumors without regional lymph node metastasis (stage I: n = 12; stage II: n = 57). Only a few cSCCs have tumor stage III or IV (n = 7) due to tumor size or regional lymph node metastases, respectively. In summary, no significant correlation between H2A.J expression and tumor stage could be observed in our sample cohort. Primary cSCC are divided into three major histologic grades based on their associated degree of nuclear atypia and keratinization. The majority of cSSCs are well differentiated, with tumor cells containing only slightly enlarged, hyperchromatic nuclei with abundant cytoplasm. They often produce large amounts of keratin, resulting in the formation of extracellular keratin beads. These well-differentiated tumors are generally associated with very low malignant potential (grade 1). In contrast, cSCC can also present as poorly differentiated tumors with grossly enlarged, pleomorphic nuclei, showing a high degree of atypia and frequent mitoses. Keratin production in these cells is significantly reduced. Typically, these poorly differentiated tumors have much more aggressive clinical behavior with increased rates of metastasis and recurrence (grade 3). Moderately differentiated subtypes exhibit features of both well-differentiated and poorly differentiated tumors (grade 2). In our tumor samples, we observed a significant correlation between H2A.J expression and tumor grading, i.e., grade 3 cSCCs are significantly more often characterized by high nuclear H2A.J expression compared to more differentiated G1 and G2 tumors. Overall, these results suggest that high nuclear H2A.J expression may be associated with more aggressive tumor behavior (Figure 5).

## 4. Discussion

In response to radiation-induced DNA damage, cell cycle checkpoints are activated to block cell cycle progression and prevent the propagation of cells with damaged DNA. However, IR exposure can induce multiple pro-survival signaling pathways acting co-jointly to reduce radiation-induced cytotoxicity and promote the development of radioresistance. In this study, we show that the overexpression of histone variant H2A.J overcomes radiation-induced senescence despite exposure to high IR doses. Transcriptome analysis following IR exposure revealed that many fundamental mechanisms essential for tumor development and progression are dysregulated by H2A.J overexpression. In addition to DNA damage response mechanisms, cell proliferation and cell cycle regulation, cytokine and cytokine receptor activity, tumor suppressors and oncogenes, and especially the EMT program were dysregulated in the irradiated H2A.J-OE fibroblasts. As part of the radiation-induced transcriptome, WNT16 was the most significantly upregulated gene in the H2A.J-overexpressing fibroblasts. Previous work already described the precise molecular mechanisms of this stress-induced WNT16 signaling pathway for the development of radioresistance and EMT [29]. Since H2A.J plays an important role in the epidermal homeostasis of human skin [40], primary cSCCs were investigated for H2A.J protein expression. Undifferentiated tumors were associated with high nuclear H2A.J expression, highlighting the oncogenic significance of H2A.J overexpression.

Hallmarks of cancer include fundamental biological capabilities acquired during multistage tumor development. These hallmark functions include not only the maintenance of proliferation potential, but also the evasion of immune destruction, reprogramming of energy metabolism, induction of angiogenesis, and activation of invasion and metastasis, summarized as the EMT complex [41]. These cancer hallmarks are promoted by genomic instability but also by epigenetic dysregulation, contributing to the acquisition of characteristic cancer features. In this sense, H2A.J overexpression could drive epigenetic reprogramming during tumorigenesis or tumor progression by altering chromatin organization and transcriptional programs and thus ultimately the functional phenotype. Histone variants are present in many types of cancer, suggesting that altered chromatin organization is closely related to cancer development [11,22]. Cancer-associated histone variants may affect the structure and stability of nucleosomes, and modified chromatin architectures with the altered recruitment of chromatin-binding proteins may dysregulate genomic DNA functions in (pre-)malignant cells [21]. The replacement of canonical histones by histone variants is dynamically regulated during radiation-induced DNA damage and may be involved in cancer development. In this experimental work, we first investigated the functional significance of H2A.J overexpression regarding the radiation response of human fibroblasts. The high degree of dissimilarity between different tumor and/or stromal cells within complex tumor microenvironments inevitably complicates data analysis and potentially confounds data interpretation. To overcome these limitations, identical fibroblast cell lines with physiological and overexpressed H2A.J levels were used to elucidate their transcriptome signatures before and after IR exposure. While normal NT fibroblasts enter permanent cell cycle arrest after IR exposure, H2A.J-OE fibroblasts overcome this radiation-induced senescence, suggesting that H2A.J overexpression results in radioresistance (Figure 1). A major obstacle to more effective cancer treatment is the ability of tumors to develop resistance to cytotoxic and cytostatic therapeutics [42]. Therefore, to explore which DNA damage-responsive pathways are involved in acquired radioresistance, gene expression profiles were analyzed comparatively in the NT and H2A.J-OE fibroblasts following the IR exposure (Figure 2, Figure 3 and Figure 4). While our previous work has already shown that H2A.J modulates the chromatin architecture and genome accessibility of key transcription factors with the modified expression of multiple SASP factors [21], the present study shows relevant differences in cell proliferation and cell cycle regulation, tumor suppressors and oncogenes, DNA damage response, as well as the EMT program. Since radiation-induced DNA damage triggers cell cycle arrest, significant differences in proliferation and cell cycle regulation were observed between the non-irradiated and irradiated fibroblasts for both NT and H2A.J-OE fibroblasts. However, our transcriptome analysis revealed that following the IR exposure, many fundamental mechanisms of cell physiology were regulated differently between the NT and H2A.J-OE fibroblasts. Particularly important in this context is that crucial cell cycle mechanisms such as cyclins and cyclin-dependent kinases are dysregulated in the H2A.J-OE fibroblasts. Our transcriptome study revealed dysregulated cyclin D1 signaling in the irradiated H2A.J-OE fibroblasts. Oncogenic signaling pathways result in uncontrolled cell cycle progression and tumor growth through the increased cyclin D1 expression (encoded by CCND1) and/or inhibition of cyclin D1 degradation [43]. Clinical studies suggest clear correlations between increased cyclin D1 expression and aggressive tumor phenotypes in cSCCs [44].

During tumor progression, oncogenic EMT functions enable cancer cells to migrate, invade, intra- and extravasate blood and lymphatic vessels [41]. Throughout the process of tumor progression, EMT functions help cells cope with changing conditions by reprogramming metabolism, improving survival through altered DNA repair, and preventing cell death, immune evasion, and improved resistance to chemo-and radiotherapy [45]. In this context, it should be emphasized that extracellular signals from fibroblasts or immune cells in the tumor microenvironment can promote dynamic EMT processes [46]. Previous studies have shown that WNT16 signaling in fibroblasts induces EMT functions in tumor cells and promotes their resistance to chemo- and radiotherapy through autocrine and paracrine signaling [28,29,47]. In addition to actual cancer cells, complex tumors contain repertoires of recruited, apparently normal cells such as cancer-associated fibroblasts and various inflammatory and immune cells that may promote malignant progression by the WNT-mediated activation of the EMT program in the tumor microenvironment [45]. However, the precise significance of radiation-induced H2A.J expression in normal compared to tumor cell populations during the development of radioresistance needs to be investigated in future studies.

Previous studies showed that the upregulation of H2A.J has oncogenic functions also in other tumors, such as breast cancer and glioblastoma multiforme (GBM) [48,49,50]. In GBM studies, the overexpression of H2A.J was linked to mesenchymal differentiation in this most aggressive brain tumor, which was identified as the driver mechanism of resistance development to chemo- and radiotherapy [50]. Since H2A.J has important functions in the epidermal homeostasis of skin [19,20,40], H2A.J expression was examined in primary cSCCs. Using chromogenic IHC, H2A.J staining patterns and expression levels were investigated by the automated analysis of TMA to obtain reproducible quantitative data that can be used to identify relevant correlations with the clinicopathological features of cSCC. Our results showed that high amounts of H2A.J+ tumor cells were detected particularly in undifferentiated G3 tumors, suggesting that nuclear H2A.J expression correlates with the differentiation status of cSCCs and may contribute to more aggressive tumor phenotypes.

## 5. Conclusions

H2A.J may be located at the intersection of complex transcriptional networks capable of modulating important pathophysiological functions in response to genotoxic stress. The epigenetic dysregulation of the H2A.J gene in response to IR can lead to altered tissue homeostasis with abnormal cell proliferation and differentiation. Future studies should clarify the precise role of H2A.J in the development of radioresistance and in the promotion of tumorigenesis and tumor progression.

## Figures and Tables

**Table 1:** Age- and gender-associated distribution of patients, as well as their anatomical localization and histologic features of their primary cSCCs.

	n	H2A.J Positive Cells % ± SEM	*p*-Value
**Age**			
Young < 50y	16	28:47 ± 8.803	0.8446
Aged ≥ 50	59	30.11 ± 3.620	
**Gender**			
Male	55	35.58 ± 8.502	0.0225
Female	20	27.64 ± 3.437	
**Sun exposure**			
exposed	56	31.03 ± 4.172	0.5227
non-exposed	19	26.01 ± 5.272	
**TNM stage**			
I	12	42.76 ± 11.64	0.0980
II	56	25.01 ± 3.35	
III	3	53.57 ± 24.46	
IV	4	39.28 ± 13.46	
**Tumor grade**			
1	42	23.20 ± 3.438	0.0001
2	23	26.74 ± 5.692	
3	10	64.21 ± 11.94	

## Data Availability

Research data are stored in an institutional repository and will be shared upon request to the corresponding author.

## Supplementary Materials

The following supporting information can be downloaded at: https://www.mdpi.com/article/10.3390/genes15070851/s1, Figure S1: Formation of SAHF following 20 Gy IR; Figure S2: Digital image analysis of H2A.J expression in cSCCs.
