# Supplementary figures and images for: Studies on Human Cultured Fibroblasts and Cutaneous Squamous Cell Carcinomas Suggest That Overexpression of Histone Variant H2A.J Promotes Radioresistance and Oncogenic Transformation
Source: Genes (Basel). 2024 Jun 27;15(7):851. doi: 10.3390/genes15070851 (PMC11275829; doi:10.3390/genes15070851)

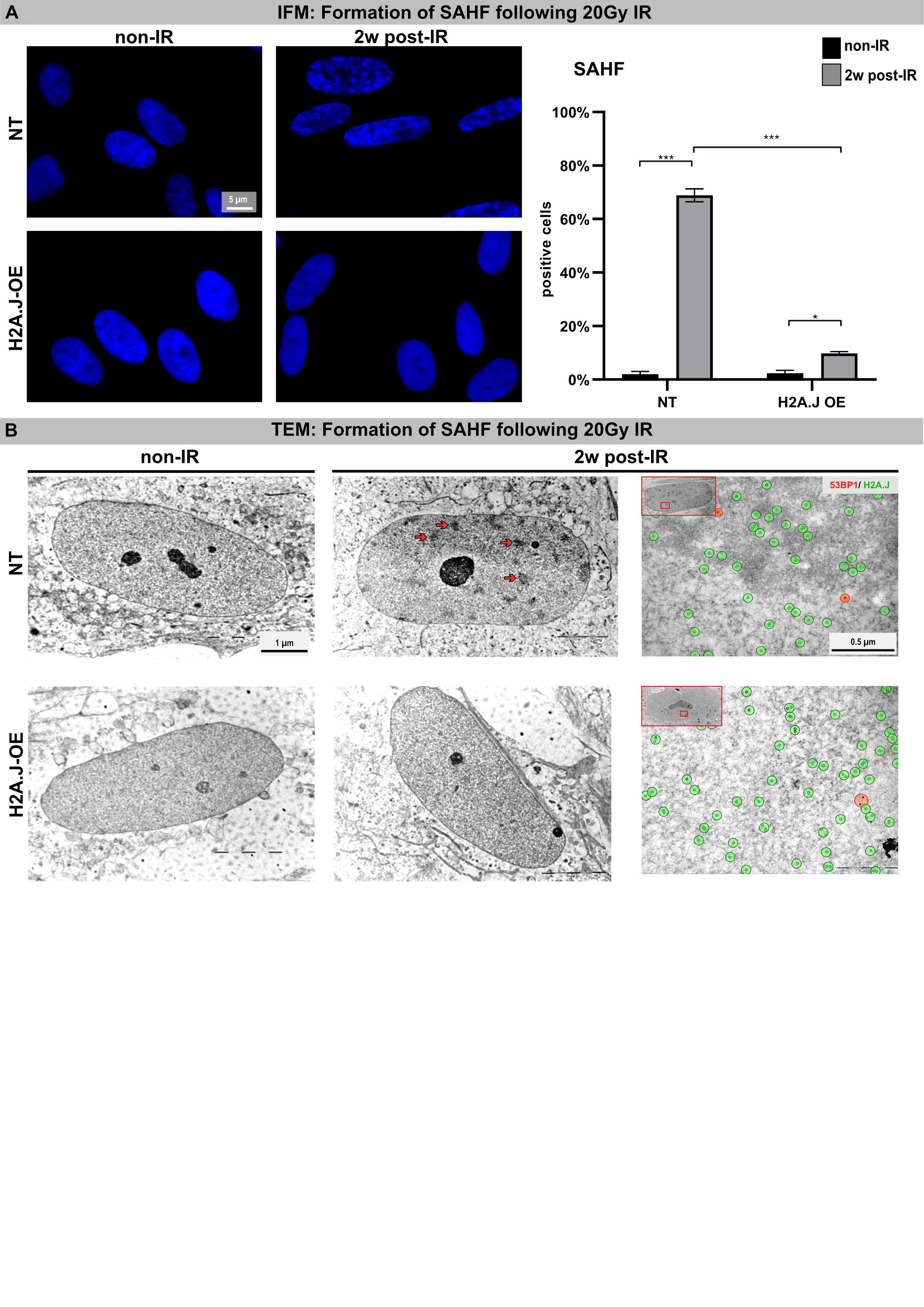

Supplement: Supplementary file 1 [file genes-15-00851-s001.zip › Figure S1 Formation of SAHF following 20 Gy IR.tiff]

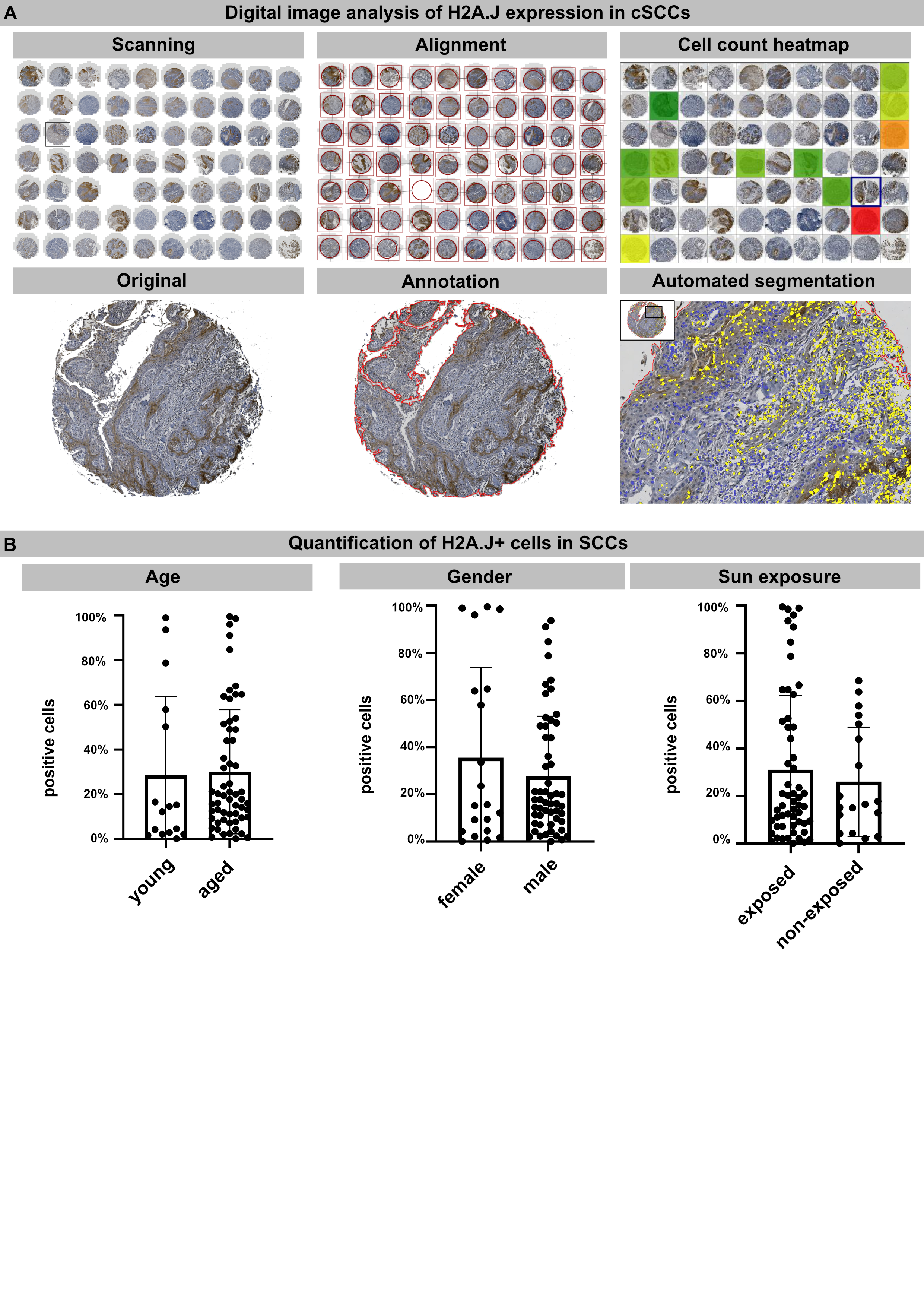

Supplement: Supplementary file 1 [file genes-15-00851-s001.zip › Figure S2 Digital image analysis of H2A.J expression in cSCCs.tiff]
